# Efficacy and Comfort Level with the Non-Sliding Lingual Orthodontic Technique (BRIUS™) Versus Labial Full Fixed Appliances: A Pilot Randomized Controlled Clinical Trial

**DOI:** 10.3390/dj12110368

**Published:** 2024-11-18

**Authors:** Mohammed Hussain Alzainal, Stephen Warunek, David A. Covell, William Tanberg, Aron Aliaga-Del Castillo, Lucia Cevidanes, Thikriat Al-Jewair

**Affiliations:** 1 Department of Orthodontics, School of Dental Medicine, State University of New York at Buffalo, Buffalo, NY 14214, USA; malzaina@buffalo.edu (M.H.A.); warunek@buffalo.edu (S.W.); dacovell@buffalo.edu (D.A.C.J.); whtanber@buffalo.edu (W.T.); 2 Department of Orthodontics, School of Dentistry, University of Michigan, Ann Arbor, MI 48104, USA; aaliagad@umich.edu (A.A.-D.C.); luciacev@umich.edu (L.C.)

**Keywords:** BRIUS, lingual, efficiency, discomfort, irregularity index

## Abstract

**Introduction:** This pilot two-arm, parallel group, randomized clinical trial aimed to compare the efficacy of tooth movement and patient comfort during orthodontic leveling and alignment with the BRIUS™ lingual system (BR) versus labial full fixed appliances (LFFAs). **Methods:** Patients in the permanent dentition with mild to moderate crowding were recruited at the University at Buffalo and randomly assigned into the BR group (seven patients) or LFFAs group (six patients). The patients’ dental arches were scanned before bonding (T1) and after 18 weeks (T2). Digital dental model superimpositions were performed to three-dimensionally evaluate tooth movement. Changes between T1 and T2 were measured in the x, y, and z planes. Little’s Irregularity Index (LII) was also assessed at T1 and T2. An electronic questionnaire was completed daily for 7 days after T1 to evaluate the patients’ comfort levels. **Results:** After 18 weeks, similar displacements were observed for all teeth in both groups except for the lower left second premolar (LL5) in the x-axis which showed greater displacement in the BR group (*p* = 0.016). Groups showed similar changes in LII. Discomfort during the first week after bonding was greater on the tongue in the BR group and on the lips and cheeks in the LFFAs group. Tongue discomfort caused by the BR lasted for around 3 days. **Conclusion:** The BR and LFFAs showed similar teeth displacements and therefore were equally effective at leveling and aligning teeth. LFFAs caused cheek- and lip-borne discomfort while the BR caused tongue-borne discomfort during the first week after bonding. Larger studies with longer follow-ups are needed to obtain more definitive results.

## 1. Introduction

Lingual orthodontic appliances have been evolving over the years owing to advancements in digital dentistry and computer-aided design and manufacturing (CAD/CAM) [1]. Some advantages of lingual appliances include enhanced esthetics and improved biomechanical control over tooth movement compared to labial appliances [2]. A relatively new lingual appliance called BRIUS™ was introduced by Mehdi Peikar in 2017 with the aim of treating patients more efficiently by minimizing side effects related to round-tripping and variable force delivery. The BR appliance incorporates four advanced features: (1) custom base non-prescription brackets with an indirect bonding protocol; (2) a pre-formed framework, referred to as an Independent Mover™ (IM) [3]; (3) independent and simultaneous teeth movement within the arch; (4) precise force delivery system with shape memory incorporated in the IM.

The IM has a customized arm for each tooth where once the arm is deflected and ligated into the lingually bonded bracket, each arm operates as a compliant or flexible mechanism and is constructed based on the projected final position of each tooth. Tooth movements resulting from the appliance are the result of the memory in the arms to return to their original shape. The development of such compliant mechanism appliances was facilitated by CAD/CAM technology, wherein the appliance is constructed with arm components having varying thicknesses tailored to the specific force requirements for tooth movement, as identified through a Finite Element Analysis (FEA). Once this appliance is affixed to the teeth in their initial alignment, each tooth progressively shifts simultaneously in all three planes of space towards its designated final position, independent of the adjacent teeth [2,3].

It is claimed that the BR can treat orthodontic cases with varying complexities, eliminating the need for post-insertion adjustments, such that a single IM engagement suffices to attain full treatment objectives for the majority of cases [4]. The absence of adjustments is claimed by the manufacturer as a contributor to enhanced patient comfort during the course of treatment [4]. The manufacturer also asserts that due to the personalized tooth movement approach, the BR facilitates accelerated and more efficient tooth movement, consequently leading to reduced treatment durations [4,5]. Given the unique biomechanics of the BR appliance and its reported benefits in efficiency and patient comfort, clinical investigations are needed to establish evidence supporting these claims.

### Specific Objectives and Hypotheses

The primary aim of this pilot randomized controlled clinical trial was to compare the efficacy of tooth movement during alignment and leveling between the BR and labial full-fixed appliances (LFFAs) in adolescents undergoing comprehensive orthodontic treatment. The secondary aim was to assess the comfort levels linked to both appliance types during the first week of initial adjustment. The null hypothesis postulated that the BR would exhibit similar efficacy of tooth movement and a similar comfort level compared to FFA in aligning and leveling teeth during orthodontic aligning and leveling.

## 2. Materials and Methods

### 2.1. Trial Design

This pilot study was conducted as a 2-arm parallel single-center randomized controlled clinical trial and followed the Consolidated Standard of Reporting Trials (CONSORT) recommendations [6]. This study was approved by the University at Buffalo Health Sciences Institutional Review Board (#00004055) and registered on the ClinicalTrials.gov Protocol Registration and Results System (# NCT04347018).

### 2.2. Participants

Subjects were recruited at the University at Buffalo School of Dental Medicine Orthodontic Clinic. The sample population consisted of subjects between 10 and 18 years of age presenting for orthodontic treatment. Subjects were included if they had Angle’s Class I or II (up to half cusp) molar relationship, fully erupted permanent dentition, mild to moderate crowding (≤7 mm), and good oral hygiene as determined by the orthodontist at each adjustment visit. Subjects were excluded if they had a previous history of orthodontic/orthognathic surgical treatments, tooth extractions, missing teeth, or showed radiographic bone loss on the dental panoramic image. Patients who presented for treatment were screened, and those that met the eligibility criteria were invited to enroll in the trial during their consultation visit. Parents/guardians and their children signed the consent and assent forms for participation in this study.

### 2.3. Interventions

The BR group was treated with the BRIUS appliance (BRIUS, Plano, TX, USA). The treatment was planned through a virtual treatment planning portal termed the BRIUS Planner™. Once the final teeth positions were determined, the BRIUS plan was approved and a set of maxillary and mandibular IMs were fabricated based on the final tooth positions. Shipped along with the IMs was an indirect bonding tray that was preloaded with non-prescription 2D^®^ Lingual brackets from Forestadent (Pforzheim, Germany).

The bonding protocol recommended by the manufacturer was as follows. Moisture control was maintained using IsoVac (Zyris, Goleta, CA, USA) during the appliance bonding procedures, including prophylaxis, sandblasting of the lingual surfaces of teeth with EtchMaster^®^ Tips (Groman Dental, Margate, FL, USA), etching with 35% phosphoric acid Ultra-Etch^®^ (Ultradent, South Jordan, UT, USA), and bonding with Assure PLUS^®^ (Reliance Orthodontics, Itasca, IL, USA). Once bonding and conditioning were complete, Rely X resin cement (3M Unitek, Monrovia, CA, USA) was loaded onto the brackets and the clear indirect bonding tray was seated onto the dental arch until the tray conformed to the shapes of all teeth. Each tooth embedded in the tray was light-cured for 15 s. The tray was then removed and the brackets were light-cured for another 15 s.

**Figure 1:**
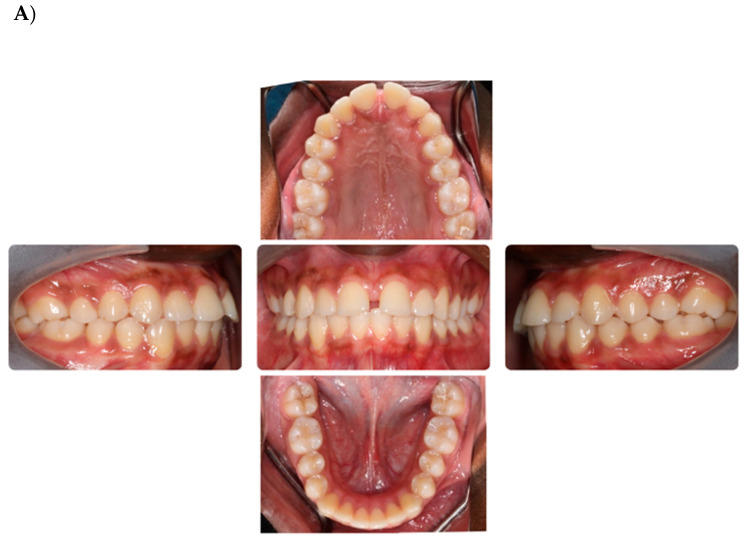

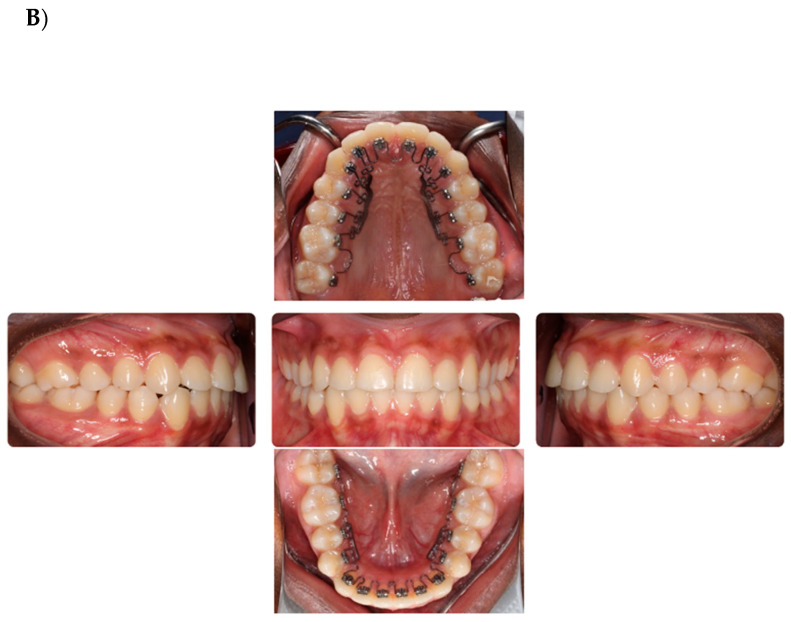
(**A**) Pre-treatment intraoral photographs (T1); (**B**) 18 weeks into treatment with BRIUS (T2).

At each visit, treatment progress, tooth movement, and bonding failures were assessed, and no adjustments were made to the appliance. Any debonded brackets were rebonded to their original position using the indirect bonding tray by cutting out a single tooth from the full arch template originally provided by the manufacturer. The bonding protocol described earlier was used for the rebonding procedures (Figure 1).

The LFFAs group underwent treatment with 0.018” slot, MBT prescription, 3M UNITEK Victory Series™ adhesive coated brackets (3M, Monrovia, CA, USA) bonded to all teeth mesial to the first molars and with bands cemented on the first molars (3M Unitek, Monrovia, CA, USA).

Archwire progression in each dental arch was 0.014”, 0.016”, and 0.16 × 22” NiTi (Ormco Corporation, Glendora, CA, USA). Each wire was in place for 6 weeks using elastomeric ligation. In cases of bracket bond failure, brackets were rebonded to the most appropriate position. Treatment in both groups was rendered by multiple providers and supervised by one faculty member.

Intraoral photographs and digital intraoral scans were obtained at the initial records appointment (T1) and at 18 weeks (T2) of treatment. Patients were scanned at the start of each visit with an iTero scanner (Align Technology, San Jose, CA, USA).

### 2.4. Outcomes

The efficacy of tooth movement was considered the primary outcome measurement. Patient comfort after initial appliance bonding was considered the secondary outcome.

(1) Efficacy of tooth movement was evaluated using 3D superimposition of digital dental models and Little’s Irregularity Index (LII).
(1.1) Digital dental model superimposition

**Figure 2:**
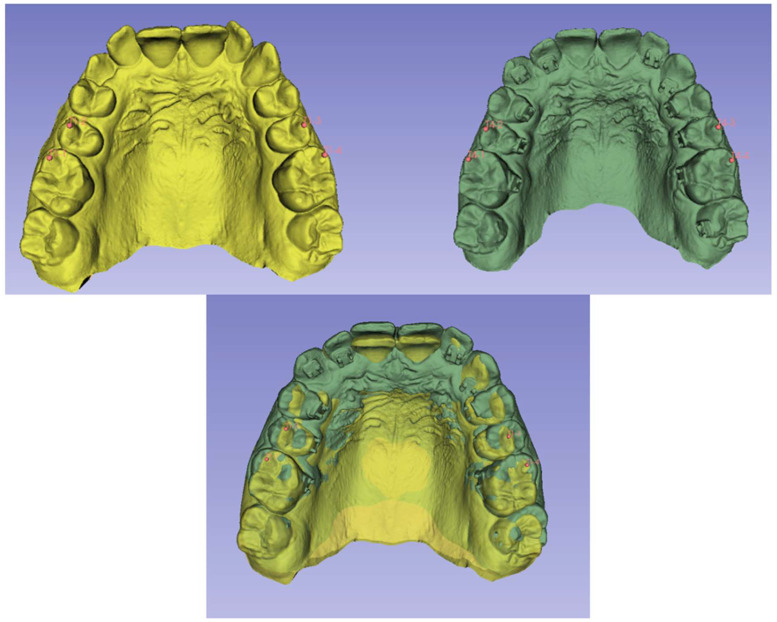
Model approximation: the mesiobuccal cusp tips of the maxillary and mandibular first molars and the buccal cusp tips of the maxillary and mandibular second premolars were selected on the T1 (yellow) and T2 (green) models. Then, a model that represented the preliminary superimposition was generated.

Three-dimensional superimposition of T1 and T2 models was performed to evaluate individual tooth movements achieved in three planes of space (x-axis [antero-posterior], y-axis [bucco-lingual], and z-axis [superior–inferior]) and combined 3D movements for each maxillary and mandibular tooth from first molar to first molar. Dental model superimpositions were performed using 3D Slicer software (version 5.6.1 for Windows, www.slicer.org). Maxillary and mandibular superimpositions, model approximations, and identification of landmarks and regions of interest were performed in reference to previous studies [7,8,9]. After uploading them into 3D Slicer software, the models were oriented within digitally constructed sagittal, axial, and coronal planes. The two models were then “approximated” using the mesiobuccal cusp tips of the first molars and the buccal cusp tips of the second premolars (Figure 2).

**Figure 3:**
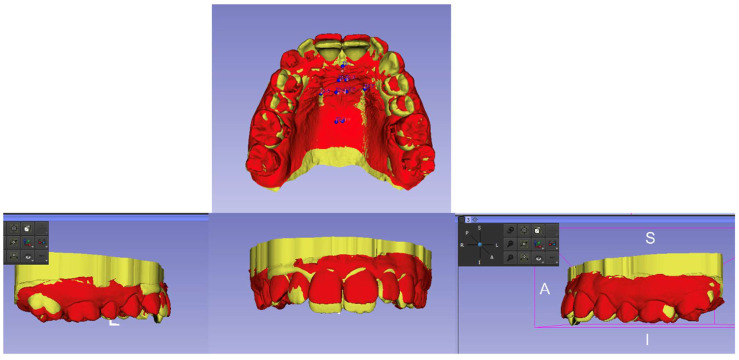
Model registration: stable landmarks (blue dots) were identified on the palate. Then, a region of interest (ROI) was created around those landmarks. The model shown in red is the registration model in the frontal, right, and left views and showing where the teeth had moved at T2.

Maxillary model superimposition was performed through the identification of landmarks and regions of interest (ROIs; Figure 3). Nine landmarks were identified on the T1 and T2 models. The landmarks were placed at the posterior-most aspect of the incisive papilla (n = 1), at the medial edges of the second palatal rugae (n = 2), at the medial and lateral edges of the third palatal rugae (n = 2), and at 10 mm posterior to the medial edges of the third palatal rugae (n = 2). Regions of interest of up to 20 mm in diameter were then created around all landmarks except the one located at the incisive papilla [7]. The T1 and T2 models were then registered using the software by matching the corresponding regions of interest. Mandibular model superimposition was performed through the placement of landmarks (a total of 10) on the mucogingival junction between the molars, between the first molar and the second premolar, between premolars, between the first premolar and the canine, and between the canine and the lateral incisor on both sides [9].

**Figure 4:**
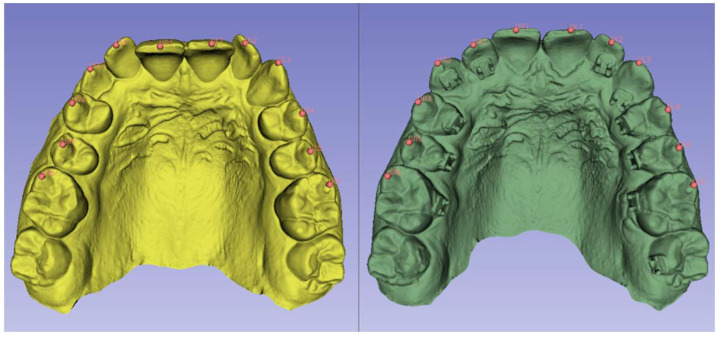
Distance quantification: landmarks were selected on each tooth from the T1 and T2 models. The landmarks were U2-2 and L2-2 (center of the incisal edge); U3 and L3 (peak of the cusp tip); U4-5 and L4-5 (peak of the buccal cusp tips); U6 and L6 (peak of the mesiobuccal cusp tips). Each landmark was recorded by tooth number, and the software quantified the distance each tooth moved in three planes of space.

For 3D movement quantification, landmarks were placed on the mesiobuccal cusp of the first molars, the buccal tip of the premolars, the cusp of the canines, and at the middle of the incisal edge of the incisors on the T1 and T2 models, and the distance between each landmark was measured (Figure 4). Once the software was programed to measure the distance between two points, the distance was automatically measured in the x, y, and z planes, as well as by an overall 3D vector.

All measurements were conducted by a single investigator (MHA) who was calibrated by an expert at using the software. Intra-examiner reliability was completed on five randomly selected models where the superimposition and measurements and LLI were repeated at least two weeks after the initial analyses.

(1.2) Little’s Irregularity Index (LII)

LLI was used to evaluate the alleviation of crowding. The index measures the alignment discrepancy in the five contact points of the lower anterior region. The scores were measured digitally by identifying the center of the mesial (CoM) and distal (CoD) anatomic contact points on each tooth. The 3D slicer software was used to measure the horizontal distance between CoM and CoD of adjacent teeth in millimeters. The scores were then compared between the BR and LFFAs groups at each time point.

(2) Patient comfort level:

Comfort level was assessed using a survey instrument developed by Wu et al. [10]. The instrument used a visual analog scale (0–10) to assess comfort of the tongue, cheeks, lips, gingiva, face, and jaw, as well as overall comfort levels [11]. It also asked whether medications were used for discomfort related to the orthodontic appliances, frequency of use, and time of the day when discomfort occurred. The survey was sent electronically to patients through QuestionPro (QuestionPro Inc., Austin, TX, USA). Each patient was instructed to complete and submit the survey daily on the first seven days after their first bonding visit (T1). The responses, expressed as percent comfort, were recorded for each participant.

### 2.5. Sample Size

Sample size was calculated based on the results of Scott et al. [12]; they determined that a standardized difference of 0.98 in the rate of tooth alignment within a period of 34 days would give a clinically relevant difference of 0.8 mm between two groups. For statistical power of 80% at a significance level of 0.05, 17 subjects were needed in each group for a total sample size of 34 subjects.

### 2.6. Randomization

Block randomization was conducted by an independent statistician uninvolved in the data collection process. The outcomes of this randomization procedure were contained within sequentially numbered opaque envelopes that were opened on the initial bonding visit to determine the group assignment for each participant.

### 2.7. Blinding

Blinding of the subjects and investigator was not possible since patients were able to recognize whether the appliance was bonded buccally or lingually.

### 2.8. Statistical Analysis

Normality was assessed using the Shapiro–Wilk test. T-tests for differences in 3D tooth movement between the BR and LFFAs groups were performed where appropriate, along with the Mann–Whitney nonparametric equivalents. The Wilcoxon signed rank test was used to calculate within-group differences in LII values between T1 and T2. Categorical discomfort and demographic comparisons between the BR and FFA groups were tested for independence using Fisher’s exact test because there were instances of low (<5) and/or zero cell counts. Continuous variables were evaluated for significance with the Mann–Whitney test. Data were analyzed using R (v4.0.4) through RStudio (v.461), and the significance level was set at 5%.

## 3. Results

### 3.1. Participant Flow

**Figure 5:**
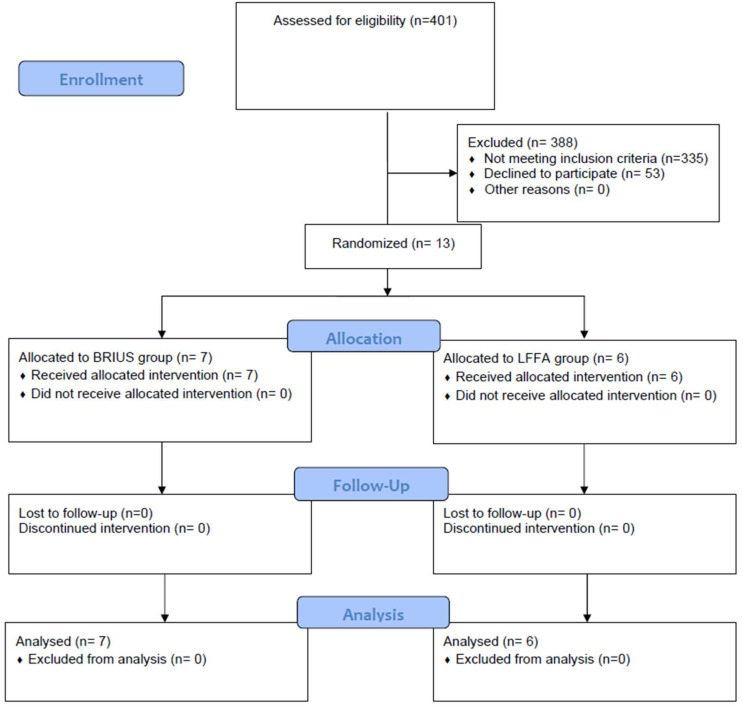
CONSORT diagram.

Patients presenting to the clinic for orthodontic treatment were screened for inclusion in this study between August 2020 and March 2022. As shown on the CONSORT diagram (Figure 5), of the 401 patients, 66 were deemed eligible to participate, and 13 of those agreed to enroll (8 males and 5 females; median age = 14.8 years; interquartile range [IQR] = 1.5; range = 12.1–17.6; Table 1). The Mann–Whitney nonparametric test showed no significant difference in the age or sex distribution between the two groups.

### 3.2. Baseline Orthodontic Characteristics

When compared at the baseline, there were no significant differences between the BR and LFFAs groups in Angle’s classification, overbite, overjet, and upper and lower dental crowding (Table 2).

### 3.3. Method Error

Dahlberg error analysis for reliability showed no significant difference in all three planes of movement, as well as combined 3D tooth movement. Similar findings were noted for LLI (intra-class correlation coefficient > 90%).

### 3.4. Extent of Tooth Movement During Leveling and Aligning Phase

Changes in the x-axis for all teeth from the upper right first molar to the lower right first molar showed no statistically significant difference between groups (Table 3). This indicates that the teeth traveled similar distances with both appliances.

Changes in the y-axis also showed no significant difference in the distance traveled, except for the LL5 (Table 4). The LL5 in the BR group showed a median total movement of 1.25 mm greater than the LFFAs group (*p* = 0.016).

There were no significant differences between the BR and LFFAs groups in distance traveled in the z-axis (Table 5) and for the overall 3D changes (Table 6).

Within-group comparisons of measurements of LII showed no significant improvement from T1 to T2 in the BR group (median difference = 1.99, IQR = 0.77, *p* = 0.125) but a significant improvement in the LFFAs group (median difference = 3.17, IQR = 1.59, *p* = 0.031; Table 7). Comparisons of changes between groups from T1 to T2 showed no significant (*p* = 0.429).

### 3.5. Discomfort Levels

Discomfort was described by patients in the BR group to be related to the teeth, gingiva, and tongue. Sporadic reports of lip or cheek pain were present as well. Discomfort perceived in the LFFAs group was related to the teeth, gingiva, and cheek and lip.

Tongue-borne discomfort (Table 8) was greater in the BR group compared to the LFFAs group, where significant differences were observed on day two, three, four, six, and seven (*p* < 0.05).

In both groups, lip-borne discomfort showed no difference during the first four days and day seven following the bonding appointment. Cheek discomfort was greater for subjects in the LFFAs group compared to the BR group for all seven days (*p* < 0.05). Gingival discomfort was not different for subjects in the two groups. There was no significant difference in medication use, frequency, or time of the day during which the discomfort was perceived.

Speech was significantly affected in the BR group compared to the LFFAs group on day two (*p* = 0.001) and three (*p* = 0.029). Sleep was not significantly different between the groups (*p* > 0.05).

### 3.6. Harms

Harms in our study included the risk of tongue pain and difficulties in maintaining oral hygiene. To minimize these risks, participants received thorough guidance and education on proper oral care practices specific to the BR.

## 4. Discussion

This pilot trial was conducted to identify whether the newly available BR orthodontic appliance can move teeth a greater distance in any or all axes during the first four months of leveling and aligning when compared to LFFAs. This study also assessed whether the BR is associated with less discomfort relative to LFFAs. No significant differences were found between the BR and LFFAs groups across all three dimensions of tooth movement (x, y, and z axes) and overall 3D measurements. Similarly, changes in LII were not significantly different within groups or between groups. These findings indicate that the BR is comparable to LFFAs during the leveling and aligning stage. Thus, the null hypothesis of no difference between appliances was accepted.

Previous studies comparing archwire-based labial and lingual fixed orthodontic appliances have yielded similar results. In a randomized clinical trial involving 20 patients with Class I malocclusion, Kaptac et al. [13] examined the alignment of the mandibular arch and reduction in LLI between customized lingual brackets and conventional labial brackets over an 18-week treatment period and observed no significant differences between the two groups. In a systematic review and meta-analysis by Ata-Ali et al. [14], the overall treatment effects of labial and lingual appliances were investigated. This review found that lingual appliances provided superior incisor torque control (less incisor proclination) when compared to labial appliances. The cephalometric values, however, showed no significant difference between the two. It is worth noting that this meta-analysis did not assess the BR appliance or provide insights into 3D tooth movements.

A literature review [15] comparing labial and lingual appliances concluded that future research should follow the CONSORT guidelines and should assess the therapeutic effects of said appliances using objective measures or therapeutic effects, including patient satisfaction, quality of life, treatment duration, and outcomes of the American Board of Orthodontics Objective Grading System, to assess occlusion. Therefore, this study evaluated the discomfort level with the BR and LFFAs during the first week after bonding. As shown by the questionnaire findings, tooth-borne discomfort was highest during the first two days following bonding in the BR and LFFAs groups, with no significant difference between the groups. This result is similar to that of previous studies on discomfort patterns following orthodontic bonding [10,15,16,17,18,19]. The current results showed that as time progressed from the bonding appointment, perceived tooth-borne discomfort subsided in both groups, reaching the lowest levels by day five. This agrees with the findings of Diddige et al. [16], who compared LFFAs to self-ligating and clear aligner appliances.

Sporadic reports of lip or cheek pain were present in the BR group, which can be related to procedural iatrogenic injury or the presence of labial buttons for antero-posterior correction of malocclusion. Most patients did not report difficulties with speech despite the lingual appliance, and there was no significant difference in speech four days after the T1 visit. This could be related to the younger age group of the samples who generally experience less discomfort during orthodontic treatment compared to adults according to several studies [20,21,22]. Current results suggest that these findings are consistent whether the new appliance is in a labial or lingual bonding position.

Gingival discomfort showed no significant difference between the two groups. Discomfort perceived in the LFFAs group had some aberrant outcomes related to tongue discomfort in the LFFAs group, and this could be related to the presence of the new intraoral appliances which can cause discomfort or ulcerations as patients move their tongue against the appliances. Wu et al. [10,19] showed that LFFAs can give patients an altered sensation of tongue position and available space, as well as perceived tongue discomfort. Based on the above findings, the hypothesis of a difference in discomfort level was rejected.

The use of medications to reduce orthodontically induced discomfort was not significantly different between the groups. This was perhaps related to the varied thresholds of discomfort amongst the study participants.

In terms of the timing of discomfort, Daguet et al. [23,24] reported that hormone release patterns during night cycles can affect discomfort perception. This finding was not supported by our study as no association was found between discomfort and time of day. Similarly, discomfort did not significantly affect sleep among the study groups. This lack of significance is consistent with other studies which showed that discomfort related to orthodontic appliances is not severe enough to affect sleep schedule [18].

Previous studies have linked orthodontic pain and discomfort to the magnitude of force being applied during orthodontic treatment [17,19,25,26,27,28]. This trial was conducted during the initial phase of orthodontic treatment when the magnitude of forces is low and continuous, which may explain the limited pain and discomfort experienced by the groups.

It was observed that because the IM exhibited a variety of loops and convoluted shapes within the arms, these tended to serve as food traps. Patients were instructed to maintain proper oral hygiene, ensuring cleanliness in these areas to prevent the accumulation of debris and adverse tissue reactions. In addition, due to the BR 2D non-prescription self-ligating brackets having a flat base and a low profile, the bonding process left up to 1 mm gaps between the flat bracket base and the convex lingual tooth contours. Those gaps had to be filled with flowable composite to minimize the risk of debonding, the accumulation of debris, and possible decalcification.

The IM showed occasional breakages, especially with the arms connected to the first and second molars. Regarding monitoring tooth movement at follow-up visits, the BR states that most mild to moderate cases can be treated with a single IM. However, if a tooth is not becoming ideally aligned, there is no indication for when the arm of an IM is no longer active other than by monitoring the progress of the case or by detaching the IM from the brackets (a somewhat cumbersome process) to determine the amount of remaining activation.

This study found that the BR appliance is equally effective as the conventional LFFAs in achieving alignment during the treatment phase. Therefore, clinicians should consider additional factors when selecting an appliance, such as cost-effectiveness and overall treatment efficiency, to optimize patient care.

Even though this study initially planned to recruit 34 patients, only 13 patients were enrolled in the study. Patients were reluctant to join the study as there was very minimal awareness about the BR appliance despite the information provided to them as part of the informed consent process. Also, many patients who qualified for the study wanted to have LFFAs for the opportunity to select the different colors of the elastomeric ligatures. Future multi-center studies are warranted to evaluate the efficiency and accuracy of the BR appliance during all treatment stages as compared to LFFAs and clear aligners. A post hoc power analysis using Little’s Irregularity Index data under an assumption of normality showed that a sample of 67 subjects per group is needed to detect statistically significant differences in future studies.

Future studies could investigate how treatment outcomes with the BR differ when planned using 3D versus 2D imaging. Additionally, research could examine the effectiveness of the BR treatment across varying degrees of malocclusion complexity, as well as in different age groups. Another area of interest would be evaluating the accuracy of the achieved BR treatment outcomes by comparing them to prescribed virtual plans.

## 5. Conclusions

The BR and LFFAs were equally effective at leveling and aligning teeth. Similar levels of discomfort were reported with both appliances during the first week after bonding. The pattern of the discomfort was tongue-borne for the BR and cheek- and lip-borne for LFFAs. Tongue discomfort caused by the BR lasted for around 3 days. Larger studies with longer follow-up durations are needed.

## Figures and Tables

**Table 1:** Sample demographics.

Variable	BRN = 7	LFFAsN = 6	Total	*p*-Value
**Age, Yrs**				
Mean (SD)	15.0 (1.9)	14.8 (3.0)	13.0	0.933 *
Median (IQR)	15.0 (2.5)	17.0 (5.0)	14.9 (2.3)	
Range (Min–Max)	12.0–17.0	11.0–17.0	15.5 (4.3)	
**Sex, N (%)**				
F	2 (15)	3 (23)	5 (38)	0.592 **
M	5 (38)	3 (23)	8 (62)	
Total	7	6 (46)	13	

* Mann–Whitney nonparametric test *p*-value; significance set at 5%. ** Fisher’s exact test for independence.

**Table 2:** Baseline orthodontic characteristics.

Variable	BR	LFFAs	Total	*p*-Value
**Right Molar Classification, N (%)**				
Class I	4 (31)	3 (23)	7 (54)	1 *
Class II	3(23)	3 (23)	6 (46)	
Total	7 (54)	6 (46)	13	
**Left Molar Classification, N (%)**				
Class I	3 (23)	4 (31)	7 (54)	0.592 *
Class II	4 (31)	2 (15)	6 (46)	
Total	7 (54)	6 (46)	13	
**Overbite (mm)**				
Mean (SD)	3.2 (2.1)	3.5 (1.7)	3.4 (1.8)	0.721 **
Median (IQR)	2.4 (3.9)	3.6 (1.8)	3.2 (3.6)	
Range (Min–Max)	1–5.6	1.1–5.8	1–5.8	
**Overjet (mm)**				
Mean (SD)	4.8 (2.6)	3.9 (1.4)	4.4 (2.1)	0.668 **
Median (IQR)	4.4 (1.1)	4.3 (1.7)	4.4 (1.2)	
Range (Min–Max)	1.1–9.0	2.1–5.7	1.1–9.0	
**Maxillary Crowding (mm)**				
Mean (SD)	2.9 (1.7)	4.1 (2.4)	3.4 (2.1)	0.520 **
Median (IQR)	2.9 (1.6)	3.8 (2.8)	2.9 (2.7)	
Range (Min–Max)	0.2–5.6	1.2–7.7	0.2–7.7	
**Mandibular Crowding (mm)**				
Mean (SD)	4.9 (2.6)	3.1(0.8)	4.1 (2.1)	0.224 **
Median (IQR)	5.5 (2.8)	3.1 (1.2)	3.8 (3.1)	
Range (Min–Max)	1.0–7.9	2.2–4.2	1.0–7.9	

* Fisher’s exact test for independence. ** Mann–Whitney nonparametric test *p*-value at a significance level of 5%.

**Table 3:** Changes in anterior–posterior (x-axis) tooth movement (mm) from T1 to T2 between BR and LFFAs groups.

Tooth	BR		LFFAs		Median Diff *	*p*-Value **
	Median	IQR	Median	IQR		
LL1	1.06	1.28	0.21	0.34	0.85	0.222
LL2	0.52	0.57	0.69	0.65	−0.17	1
LL3	0.24	0.45	0.40	0.77	−0.16	0.421
LL4	0.61	0.63	0.44	0.47	0.17	0.209
LL5	0.32	0.02	0.15	0.16	0.17	0.095
LL6	0.27	0.08	0.15	0.07	0.12	0.548
LR1	1.27	0.19	0.77	0.08	0.50	0.421
LR2	0.25	1.41	1.00	0.84	−0.75	1
LR3	0.28	0.13	0.28	0.38	0.00	0.841
LR4	0.62	0.24	0.48	0.27	0.14	1
LR5	0.40	0.26	0.21	0.32	0.19	0.310
LR6	0.50	0.37	0.72	0.62	−0.22	1
UL1	0.83	1.37	1.47	1.09	−0.64	0.841
UL2	0.12	0.45	0.98	1.62	−0.86	0.310
UL3	0.58	0.51	1.08	0.33	−0.50	0.548
UL4	1.44	0.66	0.65	0.16	0.79	0.173
UL5	1.01	0.73	0.51	0.18	0.50	0.548
UL6	0.73	1.09	0.23	0.02	0.50	1
UR1	1.64	2.35	0.99	0.72	0.65	0.841
UR2	0.67	0.34	0.76	1.69	−0.09	0.548
UR3	0.86	0.35	0.91	1.28	−0.05	0.690
UR4	0.36	0.15	0.95	0.88	−0.59	0.151
UR5	0.40	0.28	1.28	0.10	−0.88	0.032
UR6	0.34	0.10	1.37	0.92	−1.03	0.032

* A negative median difference indicates that variables in the LFFAs group moved further than the BR group by X millimeters. ** Mann–Whitney nonparametric test *p*-value at a significance level of 5%.

**Table 4:** Changes in buccal–lingual (y-axis) tooth movement (mm) from T1 to T2 between BR and LFFAs groups.

Tooth	BR		LFFAs		Median Diff *	*p*-Value **
	Median	IQR	Median	IQR		
LL1	0.27	0.09	0.15	0.19	0.12	0.310
LL2	0.26	0.98	0.27	0.14	−0.01	0.841
LL3	0.74	0.43	0.44	0.23	0.30	0.548
LL4	0.69	0.97	0.40	0.18	0.29	0.310
LL5	1.52	0.43	0.27	0.16	1.25	0.016
LL6	0.34	0.16	0.55	0.81	−0.21	1
LR1	0.13	0.03	0.20	0.43	−0.07	0.548
LR2	0.57	0.23	0.86	0.62	−0.29	0.841
LR3	0.67	0.41	0.76	0.34	−0.09	0.841
LR4	1.19	1.46	0.59	1.10	0.60	0.548
LR5	0.53	1.13	0.58	0.15	−0.05	0.69
LR6	0.24	0.29	0.41	0.27	−0.17	0.310
UL1	0.80	0.67	0.78	0.22	0.02	1
UL2	0.98	0.87	0.49	0.27	0.49	0.421
UL3	1.05	0.17	0.82	0.58	0.23	0.690
UL4	1.15	1.00	0.40	0.77	0.75	0.548
UL5	1.57	1.51	0.72	0.48	0.85	0.421
UL6	0.52	0.53	0.56	0.60	−0.04	1
UR1	0.70	1.26	0.60	0.18	0.10	0.841
UR2	0.61	1.06	0.78	0.43	−0.17	0.548
UR3	0.95	0.53	1.36	1.40	−0.41	0.421
UR4	1.01	0.59	0.81	1.89	0.20	1
UR5	0.60	1.23	0.47	1.06	0.13	0.421
UR6	0.47	0.64	0.47	0.11	0.00	0.841

* A negative median difference indicates that variables in the LFFAs group moved further than the BR group by X millimeters. ** Mann–Whitney nonparametric test *p*-value at a significance level of 5%.

**Table 5:** Changes in superior–inferior (z-axis) tooth movement (mm) from T1 to T2 between BR and LFFAs groups.

	BR		LFFAs			
Tooth	Median	IQR	Median	IQR	Median Diff *	*p*-Value **
LL1	0.37	0.07	0.56	0.69	−0.19	0.841
LL2	0.42	0.68	0.25	0.66	0.17	0.690
LL3	0.28	0.3	0.49	0.40	−0.21	1
LL4	0.21	0.09	0.37	0.51	−0.16	0.421
LL5	0.41	0.37	0.50	0.45	−0.09	1
LL6	0.16	0.19	0.27	0.26	−0.11	0.841
LR1	0.40	1.07	0.79	1.22	−0.39	0.690
LR2	0.73	0.86	0.45	0.65	0.28	0.421
LR3	0.33	0.71	0.41	0.35	−0.08	0.690
LR4	0.49	0.25	0.41	0.16	0.08	1
LR5	0.41	0.21	0.26	0.43	0.15	1
LR6	0.38	0.17	0.46	0.25	−0.08	1
UL1	1.23	1.05	0.44	0.35	0.79	0.548
UL2	0.64	0.61	1.09	0.77	−0.45	0.222
UL3	0.79	0.12	0.64	0.94	0.15	0.548
UL4	0.38	0.66	0.30	0.17	0.08	0.222
UL5	0.41	0.65	0.56	0.55	−0.15	0.690
UL6	0.75	0.25	0.52	0.07	0.23	0.310
UR1	1.23	1.65	0.73	0.69	0.50	0.841
UR2	0.58	0.38	0.49	1.30	0.09	1
UR3	1.35	1.22	0.74	0.84	0.61	1
UR4	0.28	0.04	0.24	0.87	0.04	0.841
UR5	0.43	0.16	0.56	1.09	−0.13	1
UR6	0.24	0.69	0.37	1.13	−0.13	1

* A negative median difference indicates that variables in the LFFAs group moved further than the BR group by X millimeters. ** Mann–Whitney nonparametric test *p*-value at a significance level of 5%.

**Table 6:** Changes in overall 3D tooth movement (mm) from T1 to T2 between BR and LFFAs groups.

	BR		LFFAs		Median Diff *	*p*-Value **
Tooth	Median	IQR	Median	IQR		
LL1	1.65	0.66	1.04	0.41	0.61	0.222
LL2	1.13	1.02	1.12	0.56	0.01	0.841
LL3	0.77	0.66	0.78	0.68	−0.01	0.841
LL4	1.32	0.41	1.04	0.61	0.28	0.151
LL5	1.76	0.66	0.75	0.36	1.01	0.056
LL6	0.61	0.22	0.90	0.30	−0.29	0.548
LR1	1.70	0.78	1.61	0.74	0.09	0.690
LR2	1.87	1.18	1.43	0.15	0.44	1
LR3	1.12	0.16	1.10	0.20	0.02	0.841
LR4	1.37	1.29	1.19	0.64	0.18	0.690
LR5	0.89	0.98	1.13	0.58	−0.24	0.690
LR6	0.91	0.33	0.86	1.33	0.05	0.841
UL1	1.64	0.91	1.89	1.15	−0.25	0.690
UL2	1.77	0.98	2.81	1.75	−1.04	0.421
UL3	1.48	0.45	1.96	0.81	−0.48	1
UL4	1.90	0.32	0.95	0.46	0.95	0.151
UL5	2.21	0.50	0.88	0.28	1.33	0.421
UL6	1.20	0.42	0.84	0.48	0.36	0.690
UR1	2.69	2.00	1.76	1.81	0.93	0.841
UR2	1.45	0.36	2.21	2.56	−0.76	0.421
UR3	1.86	0.57	2.36	0.56	−0.50	0.222
UR4	1.26	0.69	1.83	2.18	−0.57	0.548
UR5	1.20	1.07	1.47	1.24	−0.27	0.841
UR6	1.22	0.24	1.90	1.68	−0.68	0.421

* Negative median difference means the variable in the LFFAs group moved more than the BRIUS group by X millimeters. ** Mann–Whitney nonparametric test *p*-value at a significance level of 5%.

**Table 7:** Little’s Irregularity Index (LLI) at T1 and T2.

Time Point	T1			T2			Difference T1-T2		
LLI	BR	LFFAs	All	BR	LFFAs	All	BR	LFFAs	All
Mean (SD)	5.38 (3.04)	5.04 (1.61)	5.22 (2.40)	3.67 (1.68)	1.91 (0.9)	2.71 (1.54)	2.05 (2.35)	3.13 (1.33)	2.64 (1.85)
Median (IQR)	4.81 (4.26)	5.93 (1.98)	5.86 (2.79)	3.12 (1.92)	1.68 (1)	2.36 (1.76)	1.99 (0.77)	3.17 (1.59)	2.55 (1.90)
Range	1.78–10.00	2.51–6.27	1.78–10.00	1.71– 5.96	1.06–3.43	1.06–5.96	−1.34–5.26	1.25–4.94	−1.34–5.26
*p*-value *	0.945			0.082			0.429		

* Mann–Whitney nonparametric test; *p*-value at a significance level of 5%.

**Table 8:** Perceived discomfort at seven days after initial bonding.

		Discomfort Type															
		Tooth				Tongue			Lip			Cheek			Gingival		
Date Since Initial Bonding	Group	n	Median	IQR	*p*-Value *	Median	IQR	*p*-Value *	Median	IQR	*p*-Value *	Median	IQR	*p*-Value *	Median	IQR	*p*-Value *
Day 1	LFFAs	7	73.00	28.50	0.954	0.00	10.00	0.107	18.00	29.50	0.352	45.00	42.00	0.032	31.00	27.50	0.271
	BR	8	68.00	40.25		54.00	60.75		4.50	10.00		2.50	10.75		15.50	19.00	
Day 2	LFFAs	5	56.00	26.00	0.898	0.00	8.00	0.026	16.00	14.00	0.546	36.00	6.00	0.023	27.00	25.00	0.286
	BR	9	58.00	25.00		48.00	19.00		6.00	13.00		2.00	6.00		17.00	29.00	
Day 3	LFFAs	7	40.00	19.50	0.886	0.00	2.00	0.003	11.00	12.00	0.387	24.00	19.00	0.008	19.00	10.00	0.774
	BR	6	36.50	22.25		37.00	4.50		5.00	4.25		4.50	6.25		13.00	16.50	
Day 4	LFFAs	7	34.00	15.50	0.159	0.00	3.50	0.006	7.00	4.50	0.115	18.00	8.50	0.017	13.00	10.00	0.063
	BR	7	17.00	15.50		20.00	13.00		2.00	3.50		3.00	4.00		2.00	5.50	
Day 5	LFFAs	6	21.00	8.00	0.029	0.00	8.25	0.128	6.50	10.00	0.040	9.50	8.50	0.018	5.50	4.00	0.213
	BR	9	8.00	5.00		8.00	14.00		0.00	1.00		0.00	1.00		0.00	3.00	
Day 6	LFFAs	6	10.00	5.75	0.222	0.00	0.75	0.003	3.50	4.75	0.050	4.50	4.00	0.050	2.50	4.50	0.326
	BR	7	8.00	4.00		7.00	5.00		0.00	0.00		0.00	1.50		0.00	3.50	
Day 7	LFFAs	7	7.00	9.50	0.387	0.00	0.00	0.016	3.00	6.00	0.485	3.00	4.50	0.018	0.00	0.00	1
	BR	6	5.00	5.75		6.00	4.25		0.00	1.50		0.00	0.00		0.00	0.00	

* Mann–Whitney nonparametric test; *p*-value at a significance level of 5%.

## Data Availability

Dataset available on request from the authors.
